# Postprandial Responses to a Standardised Meal in Hypertension: The Mediatory Role of Visceral Fat Mass

**DOI:** 10.3390/nu14214499

**Published:** 2022-10-26

**Authors:** Panayiotis Louca, Sarah E. Berry, Kate Bermingham, Paul W. Franks, Jonathan Wolf, Tim D. Spector, Ana M. Valdes, Phil Chowienczyk, Cristina Menni

**Affiliations:** 1 Department of Twin Research, King’s College London, St Thomas’ Hospital Campus, London SE1 7EH, UK; 2 Department of Nutritional Sciences, King’s College London, Franklin Wilkins Building, London SE1 9NH, UK; 3 Genetic & Molecular Epidemiology Unit, Department of Clinical Sciences, Lund University, SE-20502 Malmo, Sweden; 4 Zoe, London SE1 7RW, UK; 5 Nottingham NIHR Biomedical Research Centre, School of Medicine, University of Nottingham, Nottingham NG5 1PB, UK; 6 Vascular Risk & Surgery, King’s College London, St Thomas’ Hospital Campus, London SE1 7EH, UK

**Keywords:** postprandial, hypertension, insulinaemia, triglyceridaemia, inflammation

## Abstract

Postprandial insulinaemia, triglyceridaemia and measures of inflammation are thought to be more closely associated with cardiovascular risk than fasting measures. Although hypertension is associated with altered fasting metabolism, it is unknown as to what extent postprandial lipaemic and inflammatory metabolic responses differ between hypertensive and normotensive individuals. Linear models adjusting for age, sex, body mass index (BMI), visceral fat mass (VFM) and multiple testing (false discovery rate), were used to investigate whether hypertensive cases and normotensive controls had different fasting and postprandial (in response to two standardised test meal challenges) lipaemic, glycaemic, insulinaemic, and inflammatory (glycoprotein acetylation (GlycA)) responses in 989 participants from the ZOE PREDICT-1 nutritional intervention study. Compared to normotensive controls, hypertensive individuals had significantly higher fasting and postprandial insulin, triglycerides, and markers of inflammation after adjusting for age, sex, and BMI (effect size: Beta (Standard Error) ranging from 0.17 (0.08), *p* = 0.04 for peak insulin to 0.29 (0.08), *p* = 4.4 × 10^−4^ for peak GlycA). No difference was seen for postprandial glucose. When further adjusting for VFM effects were attenuated. Causal mediation analysis suggests that 36% of the variance in postprandial insulin response and 33.8% of variance in postprandial triglyceride response were mediated by VFM. Hypertensive individuals have different postprandial insulinaemic and lipaemic responses compared to normotensive controls and this is partially mediated by visceral fat mass. Consequently, reducing VFM should be a key focus of health interventions in hypertension. **Trial registration:** The ClinicalTrials.gov registration identifier is NCT03479866.

## 1. Introduction

Hypertension is the most prevalent modifiable risk factor for cardiovascular morbidity and mortality affecting over 1.3 billion people around the world [1]. Studies have shown that hypertension clusters with metabolic factors including glucose intolerance, hyperinsulinaemia, and dyslipidaemia [2]. Indeed, results from the prospective follow-up study, Pressioni Arteriose Monitorate E Loro Associazioni (PAMELA), suggest that elevated blood pressure (BP) is the most common component of the metabolic syndrome (MetS), with 95.4% of participants with MetS having elevated BP, and up to 80% of individuals with MetS being hypertensive [3,4]. Moreover, hypertensive individuals that fulfil the criteria for MetS have up to a 73% increased age and risk factor-adjusted risk for cardiovascular events [5]. Hypertension may also be linked with the onset of new MetS [6]; it is strongly associated with insulin resistance, a component of MetS, independently of other risk factors, including obesity [7]. Mechanisms behind this may centre around the hormonal actions of insulin, which can regulate renal sodium clearance [8], a key mechanism involved in BP regulation. The increased cardiovascular risk associated with MetS and hypertension may also be linked to endothelial dysfunction and atherosclerosis [2].

Although previous research has explored fasting metabolism in hypertensive individuals, the majority of the population spend most of their waking hours in a postprandial state [9,10] and postprandial glycaemia, insulinaemia, lipaemia, and inflammation are thought to be more closely associated with cardiovascular risk than fasting levels [11], it is therefore of utmost importance to understand postprandial metabolic responses in hypertensive individuals. Hypertensive individuals have been found to have higher postprandial triglyceride levels [12], and postprandial hypertriglyceridaemia also correlates with levels of visceral adiposity [13], and causal links have been shown in murine models [14,15]. Additionally, postprandial glucose disposal in the presence of insulin resistance may promote hypertension through various atherogenic processes [2].

However, a comprehensive exploration of the fasting and postprandial differences in metabolic markers (triglycerides, insulin, glucose, and inflammation), between hypertensive and normotensive individuals, when challenged by a standardised mixed-nutrient meal, is lacking. Here, we investigate whether individuals with hypertension have a different postprandial response compared to normotensive controls. We further explore whether visceral fat mass (VFM), thought to be a key marker of glucose homeostasis and lipid metabolism, is a mediator of associations between hypertension and postprandial insulin and triglyceride response in the ZOE UK PREDICT 1 study [9]—a single-arm, randomized cross-over trial of standardized meal interventions designed to quantify and predict individual variations in postprandial responses (NCT03479866).

## 2. Materials and Methods

**Figure 1:**
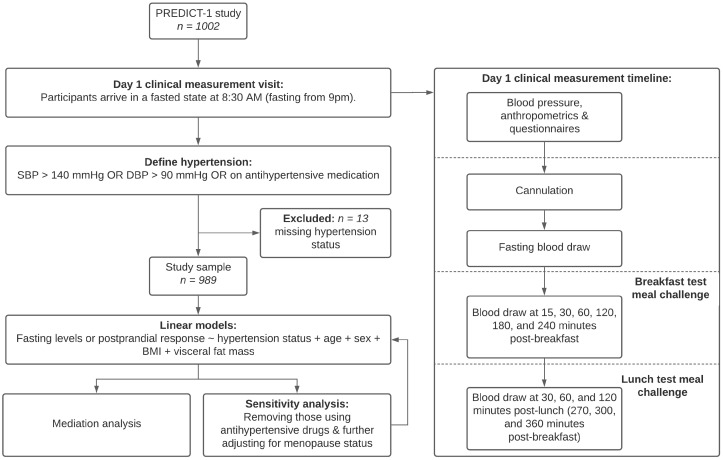
Flow chart of study analytical pipeline and clinical visit timeline. Study population and design.

A consort diagram with the study design is presented in Figure 1.

We included 989 individuals from the UK based ZOE PREDICT-1 study. The ZOE PREDICT-1 study [9] was a single-arm nutritional intervention conducted between June 2018 and May 2019. Study participants were apparently healthy individuals but included those with risk factors such as hypertension. Participants were aged between 18–65 years recruited from the TwinsUK registry [16], and the general population using online advertising. Participants attended a full day clinical visit consisting of test meal challenges followed by a 13-day home-based phase, as previously described [9].

Data relevant to this analysis pertain to the day 1 baseline clinical measurement visit at St. Thomas’ Hospital. As shown in Figure 1, during their visit, participants arrived at 8:30 am in a fasted state (fasting from 9 pm the previous night). On arrival, participants provided baseline characteristics, including age, sex, anthropometric measurements (including adiposity as described below) and BP was recorded. Participants were cannulated and a fasting blood sample was taken. Within a tightly controlled clinical setting, participants consumed meal 1: breakfast muffins and a milkshake (890 kcal, 85.5 g carbohydrate, 52.7 g fat, and 16.1 g protein at the 0-h timepoint, following baseline blood draw, BP, and anthropometrics). Venous blood samples were collected at 15, 30, 60, 120, 180, 240, 300, 360 min post meal 1. Meal 2: lunch muffins (502 kcal, 71.2 g carbohydrate, 22.2 g fat, and 9.6 g protein) was consumed at the 240-min timepoint (after the 240-min blood sample). Participants were permitted to sip water throughout (Figure 1). Outcome variables from blood sampling were blood triglyceride, glucose, insulin, and glycoprotein acetylation (GlycA) (as a marker of inflammation) levels [9]. GlycA is a particular proton nuclear magnetic resonance spectroscopy signal that reflects the methyl groups bound to N-acetylglucosamine residues attached to circulating plasma proteins and is recognised and validated as a biomarker of systemic inflammation [17]. GlycA moderately correlates with several other biomarkers of inflammation but has greater analytical precision and lower-intra-individual variability [18]. Moreover, GlycA levels have also been shown to associate with both acute and chronic inflammation, severity of inflammatory disorders, and cardiovascular events independent of other inflammatory markers [19,20]. For each of these variables, we considered (i) the baseline fasting measures; (ii) the peak (over the 6-h (360 min) visit for triglycerides and GlycA, and 2-h (240 min) for insulin and glucose) [9] and (iii) the magnitude of increase (delta increase = peak − baseline). Postprandial peaks were previously identified using line trajectories as detailed in Berry et al. 2018 [9] and the specific timepoints used here are based on these previous reports.

### 2.1. Blood Pressure

Prior to the breakfast test meal challenge, BP was measured by a trained nurse with the patient in a seated position for 3 min. The cuff was placed on the subject’s arm so that it was approximately 2–3 cm above the elbow joint of the inner arm, with the air tube lying over the brachial artery. The subject’s arm was placed on the table or supported with the palm facing upwards, so that the tab of the cuff was placed at the same level of the heart. Triplicate measurements were taken with an interval of approximately 1 min between each reading. The first reading was discarded and the mean of the second and third measurements recorded.

Participants were classified into hypertensive cases if their systolic BP ≥ 140 mmHg OR their diastolic BP ≥ 90 mmHg OR they were using antihypertensive medication, otherwise they were considered as non-hypertensive controls.

### 2.2. Adiposity

Body fat distribution was determined by whole body dual-energy X-ray absorptiometry (DXA) using a fan beam X-ray bone densitometer (QDR-4500 W, Hologic, Inc., Marlborough, MA, USA) with the participant in the supine position and analysed with QDR Systems software version 12.6 (Hologic, Inc., MA, USA), as previously described [21]. DXA fat mass from the abdominal region was recorded as VFM in a similar manner to Bertin et al. [22].

### 2.3. Statistical Analysis

Statistical analysis was performed using R version 4.0.2. Circos plot was generated using the R package ‘ggplot2’.

Continuous variables were standardised using z-scores. Linear models were used to investigate whether hypertensive cases and non-hypertensive controls had different fasting levels and postprandial responses after adjusting for age, sex, body mass index (BMI), and multiple testing (Benjamini-Hochberg false discovery rate (FDR < 0.05)). For each of these comparisons, we report effect size (Betas) and standard error (SE). To explore links between metabolic factors and visceral adiposity we conducted a sensitivity analysis by additionally adjusting for VFM.

To investigate the mediatory effects of VFM (indirect effect) in the relationship between hypertension and postprandial insulin and triglyceride levels (direct effects), we constructed a causal mediation analysis using the R package “mediation” [23]. The variance accounted for (VAF) score depicts the ratio of indirect-to-total effect and determines the proportion of the variance that can be explained by the mediator, in this instance, VFM.

We conducted additional sensitivity analyses by (i) removing any individuals on antihypertensive medication; (ii) adjusting for menopausal status; and (iii) stratifying by sex. We also investigated associations between hypertension status and insulin resistance using the Homeostasis Model Assessment for Insulin Resistance (HOMA-IR) formulated as fasting insulin (μU/mL) × fasting glucose (mmol/L)/22.5 [24].

## 3. Results

We analysed data from 989 participants who attended a full day (6-h) clinical visit consisting of two test meal challenges and had BP measurements. We included 203 hypertensive cases and 786 normotensive controls, aged 45.57 (mean, SD = 11.94) years, mainly females (72.7%), and were on average slightly overweight (BMI = 25.61, SD = 5.07) kg/m^2^ (Table 1) with an average waist-to-hip ratio of 0.85.

### 3.1. Fasting Levels

**Figure 2:**
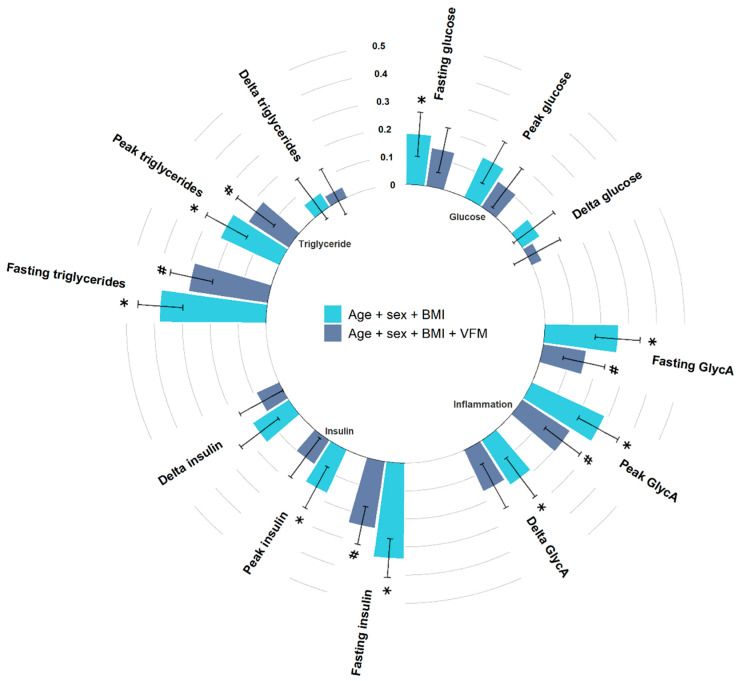
Circos bar plot with bars representing standardised coefficients of linear models between metrics and hypertension status with error bars representing standard error. Bars are colour coded based on covariates. Light blue bars indicate adjustment for age, sex, and BMI, while navy bars indicated adjustment for age, sex, BMI, and VFM. * FDR < 0.05 (for age, sex, and BMI adjusted model), # nominal significant (for age, sex, BMI, and VFM adjusted model). Abbreviations: BMI, body mass index; VFM, visceral fat mass.

We first investigated differences in the fasting (baseline) states between hypertensive cases and normotensive controls. As depicted in Figure 2, after adjusting for multiple testing we found that individuals with hypertension had significantly higher fasting glucose (Beta (SE) = 0.18 (0.08), *p* = 0.02), insulin (Beta (SE) = 0.34 (0.07), *p* = 8.61 × 10^−7^), triglycerides (Beta (SE) = 0.38 (0.08), *p* = 2.6 × 10^−6^), and GlycA (Beta (SE) = 0.26 (0.08), *p* = 1.2 × 10^−3^) (Figure 2), in line with the literature [7]. We also found that hypertensive patients have higher HOMA-IR (Beta (SE) = 0.36 (0.07), *p* = 3.6 × 10^−7^) compared to normotensive controls.

### 3.2. Peak Levels

**Figure 3:**
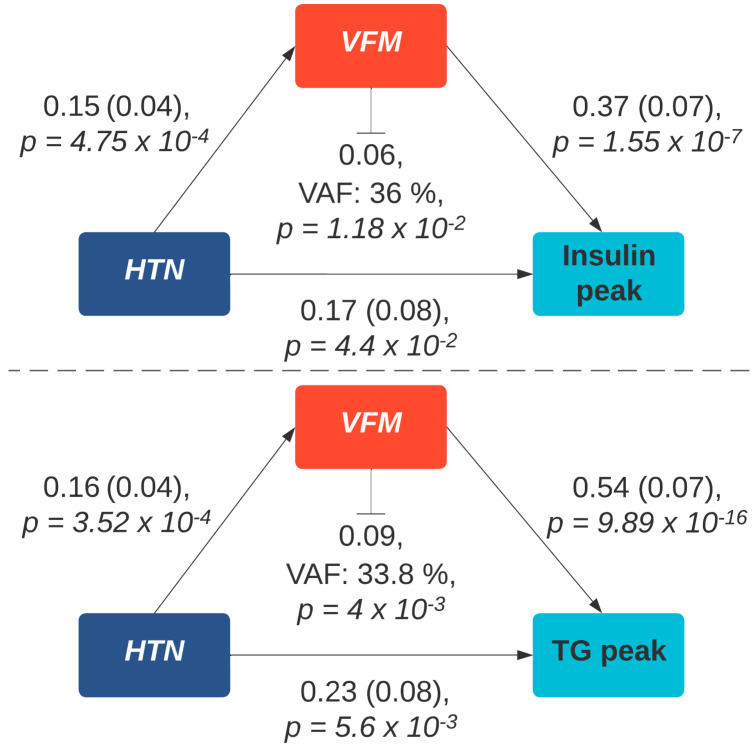
Mediation analysis of the association between hypertension and peak postprandial insulin and triglyceride response via visceral fat. Path coefficients are illustrated beside each path and indirect effect and variance accounted for score is denoted below the mediator. Abbreviations: TG, triglycerides; HTN, hypertension; VAF, variance accounted for.

We observed significantly higher postprandial peaks in insulin (2-h insulin peak, Beta (SE) = 0.17 (0.08), *p* = 4.4 × 10^−2^), triglycerides (6-h peak triglyceride, Beta (SE) = 0.23 (0.08), *p* = 5.6 × 10^−3^) and GlycA (6-h peak GlycA, Beta (SE) = 0.29 (0.08), *p* = 4.4 × 10^−4^) in hypertensive cases, after adjusting for age, sex, BMI and multiple testing (Figure 2). However, when additionally adjusting for VFM, a marker of adipose tissue strongly related to metabolic disturbances and hypertension [25], effects were attenuated (peak triglycerides, Beta (SE) = 0.17 (0.08), *p* = 0.04; peak insulin, Beta (SE) = 0.1 (0.09), *p* = 0.25); (peak GlycA, Beta (SE) = 0.2 (0.08), *p* = 0.01). Given the links between VFM and, cardiometabolic risk factors, including insulin resistance and triglycerides, we conducted a causal mediation analysis using bootstrapping and 1000 simulations to determine the indirect effect of VFM on the relationship between hypertension and postprandial triglyceride, and insulin responses. This suggested that VFM was fully mediating the positive association between hypertension and 2-h peak insulin (VAF = 36% (0.02, 0.1) *p* = 0.002), and partially mediating the positive association between hypertension and 6-h triglyceride peak (variance accounted for (VAF) = 33.8% (0.03, 0.16) *p*= 0.004) (Figure 3).

### 3.3. Change from Fasting Levels

Results were consistent when we rerun the analyses investigating the correlation between hypertension status and postprandial metabolic changes measured as delta. Beta coefficients were in the same direction, although *p* values were attenuated (Figure 2). 

### 3.4. Sensitivity Analysis

To account for potential confounding, we conducted sensitivity analyses by (i) excluding those on antihypertensive drugs, (ii) adjusting for menopausal status (pre-, peri- and post-menopausal, as determined by a health and lifestyle questionnaire), (iii) stratifying by sex. Results/effect sizes remained consistent. See Supplementary Table S1.

## 4. Discussion

In the largest study of its kind to look at differential postprandial responses in hypertensive individuals compared to normotensive subjects, we find that in addition to a disrupted fasting metabolic state, individuals with hypertension have higher postprandial insulin, triglycerides, and inflammatory responses after adjusting for traditional risk factors. Causal mediation analysis further suggests that VFM, a key risk factor in metabolic syndrome [26], fully mediates the associations between hypertension and insulin responses and partially mediates that with postprandial triglyceride response. Moreover, we find an increased level of insulin resistance in hypertensive participants compared to normotensive controls.

Postprandial lipaemia is a greater predictor of cardiovascular events, in contrast to fasting triglyceride levels [11,27]. Here, we report higher spikes in postprandial triglycerides in hypertensive cases, after consumption of a mixed-nutrient challenge. In support of our findings, Hwu et al. [12] found higher postprandial triglycerides in hypertensive participants 4-h after a 1000 kcal, high fat meal (65.9% fat, 18.9%, carbohydrates, 15.2% protein). Moreover, when compared to normotensive participants, Kolovou and colleagues [28] also report higher postprandial triglycerides in 25 individuals with essential hypertension following a meal dense in fat (83.5% fat, 14% carbohydrates, 2.5% protein). Although, in contrast to our study, the hypertensive participants were found to have normal fasting triglycerides [28]. Moreover, Kolovou et al. show significant positive correlations between BMI and maximal postprandial triglyceride concentration within the hypertensive participants. Extending this link between elevated postprandial triglycerides, hypertension, and body fat. Here, we find that VFM partially mediates the relationship between hypertension status and postprandial lipaemia, suggesting that despite the link with cardiovascular events [9], the lipaemia is likely to have a smaller effect on blood pressure than previously thought. Rather, this data suggests that the relevance of triglyceride metabolism in hypertension lies mainly in higher adipose fat. A possible explanation, is the variety of vasoactive factors (both vasodilators such as leptin, adiponectin, apelin and omectin) and vasoconstrictors like resistin, chemerin, and visfatin released by adipocytes [25] and that levels of visceral fat are directly involved in blood pressure regulation or influence blood pressure through activation of sympathetic nervous system activity [25].

Approximately, half of all patients with essential hypertension are thought to be insulin-resistant [2,29]. Indeed, here we report that hypertensive individuals have higher levels of insulin resistance, as determined by the HOMA-IR index. As expected, we also find hypertensive individuals to have postprandial hyperinsulinaemia. The links between hyperinsulinaemia and hypertension is thought to be driven via a few key mechanisms, (i) a decrease in insulin sensitivity, (ii) insulin mediated glucose disposal [2], both of which are thought to promote hypertension and atherogenesis. (iii) increased plasma aldosterone levels [30], and (iv) upregulated angiotensin II receptors [31], two components of the renin-angiotensin-aldosterone system, a critical regulator of BP. Visceral fat, particularly that deposited around the liver has been linked with impaired insulin clearance or hepatic insulin action [32], which would further exacerbate these actions, and may explain the mediation effects we observe.

Additionally, evidence of a causal role of VFM in insulin and triglyceride actions has also been found in murine models, where the removal of VFM restored insulin action and improved lipid profiles [14,15], which supports our reports of a strong mediatory role of VFM in postprandial response.

We also observe higher fasting glucose concentrations in hypertensive cases. However, despite our findings on insulin resistance, fasting glucose, and fasting/postprandial hyperinsulinaemia, we did not find significant differences in glycaemic responses. In contrast, in a cross-sectional, longitudinal analysis of 3437 individuals, of which 497 developed hypertension, fasting and postprandial glucose were independent predictors of incident hypertension [33]. This may be explained by an early stage of insulin resistance in the hypertensive cases (HOMA-IR = 1.9, Table 1), whereas hyperglycaemia is thought to become prevalent at more advanced stages [2]. 

These results suggest that hypertensive individuals may be more prone to cardiovascular events as a result of exacerbated metabolic responses. Regardless of fasting levels, an exacerbated postprandial increase in insulin, triglycerides, glucose and inflammation have detrimental effects on vascular health [26]. Postprandial hyperlipaemia has been linked with impaired lipid metabolism, endothelial dysfunction, hypercoagulability, all of which are key factors involved in atherogenesis [34]. Detrimental effects of postprandial hyperinsulinaemia relate to the hormonal action of insulin, which has the capacity to stimulate numerous cellular responses and has been shown to promote protein synthesis, de novo lipogenesis, and cellular proliferation while inhibiting autophagy, and lipolysis, necessary actions for cellular turnover. Likewise systemic inflammation is independently linked with atherogenesis and coronary heart disease events [12].

Our findings also suggest that BMI is unable to capture the true effects brought about by adiposity. Although BMI is easy to measure in contrast to VFM, the utility of BMI to distinguish between fat and muscle has long been questioned [35]. The present results suggest visceral fat mass should be more routinely measured and used as an actionable target with dietary efforts seeking to reduce visceral fat in hypertensive patients, and in turn mitigate adverse postprandial responses.

The finding that VFM is a causal mediator between hypertension and an atherogenic postprandial triglyceride response has a number of implications for management of hypertension. Firstly, it emphasises the importance of VFM and suggests that VFM should be measured and actioned as a target for treatment. Many lifestyle interventions that are advocated for hypertension, for example, weight loss and reductions in alcohol intake are expected to reduce VFM as well as hypertension but in the context of hypertension they tend to be evaluated according to the reduction in blood pressure achieved. The present results suggest that normalisation of both blood pressure and VFM is likely to achieve optimal risk prevention. Interventions such as bariatric surgery may be very effective in reducing both blood pressure and VFM [36] and their benefit in terms of reduction in VFM may strengthen this indication. The relative benefits and risk of such an intervention can, however, only be rigorously assessed by randomised clinical trials. Secondly, the importance of VFM as contributing to atherogenic risk in hypertension raises the possibility that it could be incorporated in a risk score guiding the indication for statin therapy to offset the atherogenic risk. Again, this would need to be guided by prospective studies evaluating cardiovascular risk and would require more widely available measures of VFM.

Although our study is strengthened by numerous factors, including the tightly controlled nature of the study, there are important limitations. These include (i) the use of office blood pressure measured on a single day to define hypertension, which is prone to measurement error, and white coat effect, i.e., BP increases due to physiological changes when in the presence of a clinician [37], which may have resulted in misclassification bias. These limitations can be overcome by using other means of BP measurement such as ambulatory BP monitoring. However, ambulatory BP was not available for the full sample due to the associated costs and participant burden. (ii) the predominantly female (72.5%) sex of our sample; further research may be required to accurately elucidate any differences between the sexes. (iii) Our sensitivity analysis with menopause status was based on a self-reported questionnaire rather than hormone profiles and may lack accuracy in identifying those pre- and post-menopausal. (iv) While fasting metabolic levels have been widely explored in hypertensive individuals, there is a lacuna of research administering meal challenges in individuals with hypertension and measuring postprandial metabolic responses. Accordingly, there is relative novelty in our study and a lack of studies to compare our findings to.

## 5. Conclusions

Our findings further the clinical perspective of hypertension as a metabolic disorder and suggest that visceral adiposity is a key factor exacerbating postprandial hypertriglyceridaemia and hyperinsulinaemia. Consequently, reducing VFM should be a key focus of health interventions in hypertension.

## Figures and Tables

**Table 1:** Demographic characteristics of the study population overall and by hypertension status.

	Overall (*n* = 989)		Hypertensive Cases (*n* = 203)		Normotensive Controls (*n* = 786)	
	*n*	%	*n*	%	*n*	%
**Antihypertensive drug use**	56	5.7	56	27.6	0	0
**Females**	719	72.7	135	66.5	584	74.3
**Peri-menopausal**	54	8.8	15	12.5	39	7.9
**Post-menopausal**	201	32.7	72	60	129	26.1
	***Mean***	***Sd***	***Mean***	***Sd***	***Mean***	***Sd***
**Age (years)**	45.6	11.9	52	10.2	43.9	11.8
**BMI (kg/m^2^)**	25.6	5.1	27.4	5.6	25.2	4.8
**Waist to hip ratio**	0.85	0.08	0.88	0.09	0.84	0.08
**VFM (g)**	527.2	311.6	689.8	358.8	485.1	283.7
**HOMA-IR**	1.4	1.1	1.9	1.8	1.3	0.9

Abbreviations: BMI, body mass index; VFM, visceral fat mass; HOMA-IR, Homeostasis Model Assessment for Insulin Resistance.

## Data Availability

The data used in this study are held by the department of Twin Research at King’s College London. The data can be released to bona fide researchers using our normal procedures overseen by the Wellcome Trust and its guidelines as part of our core funding (https://twinsuk.ac.uk/resources-for-researchers/access-our-data/).

## Supplementary Materials

The following supporting information can be downloaded at: https://www.mdpi.com/article/10.3390/nu14214499/s1, Supplementary Table S1: Sensitivity analysis of fasting, and postprandial metabolic responses between hypertensive cases and controls, including overall results adjusted for age, sex, and BMI; removing those using antihypertensives; further adjusting for menopause; and when stratifying by sex.
