# Supplementary material for: Postprandial Responses to a Standardised Meal in Hypertension: The Mediatory Role of Visceral Fat Mass
Source: Nutrients. 2022 Oct 26;14(21):4499. doi: 10.3390/nu14214499 (PMC9655022; doi:10.3390/nu14214499)
Supplement: Supplementary file 1 [file nutrients-14-04499-s001.zip › nutrients-1949933-supplementary.pdf]

Postprandial responses to a standardised meal in hypertension: the mediatory role of visceral fat mass

Panayiotis Louca[1], Sarah E. Berry[2], Kate Bermingham[1, 2], Paul W. Franks[3], Jonathan Wolf[4], Tim D. Spector[1], Ana M. Valdes[5], Phil Chowienczyk[6]*, Cristina Menni[1]*

[1]Department of Twin Research, King's College London, St Thomas' Hospital Campus, London SE1 7EH

[2] Department of Nutritional Sciences, King's College London, Franklin Wilkins Building, SE1 9NH London.

[3] Genetic & Molecular Epidemiology Unit, Department of Clinical Sciences, Lund University, Malmo, SE-20502, Sweden.

[4] Zoe Global Limited, London SE1 7RW, UK.

[5] Nottingham NIHR Biomedical Research Centre at the School of Medicine, University of Nottingham, Nottingham, NG5 1PB, UK.

[6] Vascular Risk & Surgery, King's College London, St Thomas' Hospital Campus, London SE1 7EH.

* denotes equal contribution.

Corresponding author:

Dr. Cristina Menni.

Department of Twin Research, King's College London, St Thomas' Hospital Campus, Westminster Bridge Road, London SE1 7EH, UK

Phone: +44 (0) 207 188 7188 (ext. 52594); email: cristina.menni@kcl.ac.uk

Supplementary Table S1. Sensitivity analysis of fasting, and postprandial metabolic responses between hypertensive cases and controls, including overall results adjusted for age, sex, and BMI; removing those using antihypertensives; further adjusting for menopause; and when stratifying by sex.

		Overall (age, sex, BMI adjusted)		Removing those on antihypertensive mediciation		Age, sex, BMI and menopausal status adjusted		Females only (*n* = 719)		Males only (*n* = 270)	
**Trait**	**Metric**	**Beta**	**SE**	**Beta**	**SE**	**Beta**	**SE**	**Beta**	**SE**	**Beta**	**SE**
**Glucose**	Baseline	0.18	0.08	0.14	0.09	0.17	0.08	0.17	0.10	0.21	0.14
	Peak	0.15	0.08	0.19	0.09	0.13	0.08	0.20	0.10	0.07	0.15
	Delta	0.07	0.09	0.13	0.10	0.05	0.09	0.12	0.11	-0.04	0.16
**GlycA**	Baseline	0.26	0.08	0.27	0.09	0.25	0.08	0.24	0.10	0.34	0.15
	Peak	0.29	0.08	0.25	0.09	0.27	0.08	0.29	0.10	0.30	0.16
	Delta	0.19	0.09	0.11	0.10	0.17	0.09	0.20	0.10	0.17	0.16
**Insulin**	Baseline	0.34	0.07	0.30	0.08	0.34	0.07	0.30	0.08	0.43	0.12
	Peak	0.17	0.08	0.12	0.09	0.16	0.08	0.11	0.10	0.37	0.14
	Delta	0.15	0.08	0.11	0.09	0.14	0.08	0.09	0.10	0.36	0.14
**Triglycerides**	Baseline	0.38	0.08	0.41	0.09	0.38	0.08	0.43	0.10	0.31	0.15
	Peak	0.23	0.08	0.18	0.09	0.22	0.08	0.29	0.10	0.16	0.15
	Delta	0.06	0.09	-0.01	0.10	0.04	0.09	0.07	0.11	0.03	0.16
